# Human Indoor Exposure to Airborne Halogenated Flame Retardants: Influence of Airborne Particle Size

**DOI:** 10.3390/ijerph14050507

**Published:** 2017-05-09

**Authors:** Mark J. La Guardia, Erika D. Schreder, Nancy Uding, Robert C. Hale

**Affiliations:** 1 College of William & Mary, Virginia Institute of Marine Science, P.O. Box 1346, Gloucester Point, VA 23062, USA; hale@vims.edu; 2 Toxic-Free Future, 4649 Sunnyside Ave N, Suite 540, Seattle, WA 98103, USA; eschreder@watoxics.org (E.D.S.); nuding@toxicfreefuture.org (N.U.)

**Keywords:** flame retardants, particle size, indoor air quality, human exposure, polyurethane, bioavailability

## Abstract

Inhalation of halogenated flame-retardants (HFRs) released from consumer products is an important route of exposure. However, not all airborne HFRs are respirable, and thus interact with vascular membranes within the gas exchange (alveolar) region of the lung. HFRs associated with large (>4 µm), inhalable airborne particulates are trapped on the mucosal lining of the respiratory tract and then are expelled or swallowed. The latter may contribute to internal exposure via desorption from particles in the digestive tract. Exposures may also be underestimated if personal activities that re-suspend particles into the breathing zone are not taken into account. Here, samples were collected using personal air samplers, clipped to the participants’ shirt collars (n = 18). We observed that the larger, inhalable air particulates carried the bulk (>92%) of HFRs. HFRs detected included those removed from commerce (i.e., polybrominated diphenyl ethers (Penta-BDEs: BDE-47, -85, -100, -99, and -153)), their replacements; e.g., 2-ethylhexyl 2,3,4,5-tetrabromobenzoate (TBB or EH-TBB); bis(2-ethylhexyl) 3,4,5,6-tetrabromophthalate (TBPH or BEH-TEBP) and long-produced chlorinated organophosphate-FRs (ClOPFRs): tris(2-chloroethyl)phosphate (TCEP), tris(1-chloro-2-propyl)phosphate (TCPP or TCIPP), and tris(1,3-dichloro-2-propyl)phosphate (TDCPP or TDCIPP). Our findings suggest estimates relying on a single exposure route, i.e., alveolar gas exchange, may not accurately estimate HFR internal dosage, as they ignore contributions from larger inhalable particulates that enter the digestive tract. Consideration of the fate and bioavailability of these larger particulates resulted in higher dosage estimates for HFRs with log *K_oa_* < 12 (i.e., Penta-BDEs and ClOPFRs) and lower estimates for those with log *K_oa_* > 12 (i.e., TBB and TBPH) compared to the alveolar route exposure alone. Of those HFRs examined, the most significant effect was the lower estimate by 41% for TBPH. The bulk of TBPH uptake from inhaled particles was estimated to be through the digestive tract, with lower bioavailability. We compared inhalation exposure estimates to chronic oral reference doses (R*f*Ds) established by several regulatory agencies. The U.S. Environmental Protection Agency (EPA) R*f*D levels for several HFRs are considered outdated; however, BDE-99 levels exceeded those suggested by the Dutch National Institute for Public Health and the Environment (RIVM) by up to 26 times. These findings indicate that contributions and bioavailability of respirable and inhalable airborne particulates should both be considered in future risk assessments.

## 1. Introduction

Halogenated flame-retardants (HFRs), such as polybrominated diphenyl ethers (PBDEs), are used in a wide array of polymer-containing consumer products, and have been widely detected in indoor environments (e.g., air, dust, and surface films) [1,2,3]. To meet Californian (USA) 1975 home furniture flammability standards (Technical Bulletin 117 or TB 117 [4]), the Penta-BDE technical formulation, formally marketed as DE-71 (Great Lakes Chemical Corp. now Chemtura Corp., Philadelphia, PA, USA) and Bromkal 70-5DE (Chemische Fabrik Kalk, Köln, Germany), among others, were added to furniture padding (polyurethane foam) to slow ignition and the spread of household fire. These Penta-BDE formulations contained PBDEs with primarily 4–6 bromines [5]. Due to human and environmental health concerns, PBDEs of the Penta- and Octa-BDE technical formulations were added in May 2009 to the Persistent Organic Pollutants (POPs) list of the Stockholm Convention, restricting their usage globally in signatory countries [6]. However, PBDEs will continue to be released into the environment from products with long lifetimes such as furniture and automobiles. Recycled materials may also reintroduce PBDEs. For example, carpet underlayment (known as rebond) may contain up to 1000 ppm (0.1% by weight) of Penta-BDEs [7]. Usage restrictions have led to the introduction of alternative halogen-containing flame retardants, including 2-ethylhexyl 2,3,4,5-tetrabromobenzoate (TBB or EH-TBB) and bis(2-ethylhexyl) 3,4,5,6-tetrabromophthalate (TBPH or BEH-TEBP). Usage of chlorinated organophosphate flame retardants (ClOPFRs) also increased, including tris(1-chloro-2-propyl)phosphate (TCPP or TCIPP) and its analog tris(1,3-dichloro-2-propyl)phosphate (TDCPP or TDCIPP). TDCPP is a suspected mutagen, formerly used in children’s sleepwear until its voluntarily withdrawal in 1977 [8]. (The nomenclature for HFRs is listed within the Supplementary Materials Table S1). These and other HFRs, some lacking comprehensive toxicity testing, have now been detected in homes and other indoor environments [9].

Partitioning of HFRs between the gas and particle-phases has been described by the gas/particle partition coefficient, *K*_p_ (m^3^·µg^−1^), (*K*_p_ = (*C*_p_/*C*_TSP_)/*C*_g_), where *C*_p_ and *C*_g_ are the organic compound concentrations (pg·m^−3^) in the particle and gas phases, respectively, and *C*_TSP_ is the concentration of total suspended particles in the air (µg·m^−3^). Cetin et al. (2008) [10] observed a strong correlation (*R*^2^ = 0.70–0.98) between log *K*_p_ and log octanol-air partitioning coefficient (*K_oa_*) for several PBDEs on airborne organic matter. Using the *K_oa_*-based equilibrium model, Cetin et al. (2008) observed at indoor temperatures (25 °C) that 2,2’,4,4’-tetrabromodiphenyl ether (BDE-47) (log *K_oa_* 10.5), with 4 bromines, reaches equilibrium within 100 days with >80% residing in the particle phase at equilibrium. In contrast, PBDEs with >5 bromines (including the fully brominated diphenyl ether, BDE-209, log *K_oa_* 18.4) require 100–1000 days, with >95% residing in the particle phase. Several additional adsorption models (i.e., Mackey and Junge-Pankow) have also described atmospheric gas-particle partitioning and suggest HFRs with a log *K_oa_* > 11 have a greater tendency to reside on organic particulates (HFRs log *K_oa_* and their partitioning coefficients are listed on Table S1). However, An et al. (2011) [11] observed that the Junge-Pankow model underestimated the sorption of most PBDE congeners in indoor air samples collected at electronic waste dismantling and incineration plants. They reported that PBDE air concentrations during working hours were 25 times higher than during non-working hours. Allen et al. (2007) [12] observed clouds of suspended particles generated by office workers’ activities (e.g., walking), resulting in greater air levels of PBDEs. Using exposure monitors affixed to individuals, Ferro et al. (1999) [13] observed that normal human activities increased levels of airborne particle matter (PM) >10 µm by 6–11 times and coarse PM (2.5–10 µm) by 2–11 times, compared to stationary indoor monitors during active periods. They observed little difference in levels of fine PM (<2.5 µm) [13,14].

Larger particles may also carry higher HFR burdens, associated with polymer fragments. Polymers often contain percent by weight levels of HFR additives. Using a stainless-steel test chamber, Rauert and Harrad (2015) [15] investigated PBDE transfers from source materials (i.e., plastic television casings containing penta-BDEs, 2,2′,3,4,4′,5′,6-heptabromodiphenyl ether (BDE-183) and BDE-209). They observed no detectable transfers to dust by volatilization at 22 °C and 60 °C, but did detect transfer from fragments abraded following motion that mimicked repeated wiping/moving/bumping of the source product. Large dust particles, approximately 30 µm in diameter, examined by electron microscopy revealed that BDE-209, having a log *K_oa_* of 18.4, was highly associated (1000 ppm or 0.1% by weight) with polymer/organic matrix, suggesting fragments of HFR-treated products are transferred to dust [16]. Hale et al. (2002) [17] reported that Penta-BDE-containing polyurethane became brittle and disintegrated within four weeks of exposure to direct sunlight. These observations, along with partitioning models, suggest that polymer additives with log *K_oa_* > 10 are less likely to volatilize at room temperature. Once incorporated into carbon-rich organic polymers of consumer products, such HFRs are more likely to remain within this matrix (e.g., polyurethane foam). However, as these polymer products fragment, HFR-containing particulates are released to the indoor environment.

Dietary dust intake via incidental ingestion is widely accepted as the major exposure route for HFRs for most individuals [18,19,20]. However, air inhalation and dermal absorption are also contributing paths [21,22]. Schreder et al. (2016) [23] estimated inhalation and dust intake exposure of several HFRs for adults and children and observed “exposure may be much higher—by one or more orders of magnitude—than previously believed based on dust ingestion as a primary exposure route” (p. 503). However, total inhalation exposure can be underestimated if the route of contaminant uptake, i.e., alveolar uptake of volatile compounds or uptake via the digestive tract of larger airborne particulates inhaled and swallowed, and the bioavailability of the contaminant dictated by the route of uptake are not taken into account. La Guardia and Hale (2015) [24] and Schreder et al. (2016) [23] detected Penta-BDEs, TBB, TBPH, and ClOPFRs, used to flame-retard polyurethane foam, in indoor dust and air samples. Indoor environments examined included gymnasiums at gymnastics facilities, with their high use of polyurethane foam products to protect against impact injuries, residences of the gymnastics coaches working within these gymnasiums, and a combination of residences, offices, and other locations. Both studies were conducted in the greater Seattle, WA, USA area. All samples were collected using personal exposure monitors, clipped to the participants’ shirt collars. This allowed air collection within the participants’ breathing zone while they were engaged in daily activities. Two air particulate fractions were obtained with each sample: (1) inhalable particulates (>4 µm, nominal) which deposit primarily in the upper respiratory tract and, (2) respirable air particulates (<4 µm, nominal) which can penetrate deep inside the lung’s gas-exchange regions. Both studies included the same HFRs. These display a wide range of log *K_oa_* values (5.31 to 16.9, Table S1) which may dictate the particle size tendency and therefore zone of deposition within the respiratory tract.

To expand our understanding of human exposure to airborne HFRs, both data sets (La Guardia and Hale, 2015 [24] and Schreder et al., 2016 [23]) were further analyzed and HFR air particulate levels within each environment were compared. Air particulate fractions (respirable and inhalable) for each HFR were evaluated and total inhalation uptake through the alveolar region of the lung and digestive tract was estimated. These estimates were then compared to total alveolar uptake. Total daily inhalation HFR exposure rates were evaluated and compared to established chronic reference dose (R*f*D) values. Finally, we discuss the human exposure implications of the air fraction exposure model for airborne HFRs in indoor environments.

## 2. Materials and Methods

### 2.1. Sampling Demographics

A total of 18 indoor environments were sampled. These included 14 common indoor spaces (i.e., residence/office (n = 10) [23], four coach residences, and four gymnasiums [24]). The gymnasiums contained substantial amounts of flame retardant-treated polyurethane foam, previously associated with increased gymnast PBDE blood levels [20]. Adults (n = 10), primarily office workers, were recruited to collect residence/office/“other” air samples during a 24-h day while engaged in normal activities (e.g., at home, work, traveling to and from home and work, shopping, socializing, and during sleep). Personal samplers (see Section 2.2. Air Sampling Methods) were worn at breathing zone level. Duration of collection ranged from 12.9 to 24.6 h, and air volumes ranged from 1.55 to 2.95 m^3^. Four coaches at four separate gymnasiums were recruited to collect 8-h air samples during a normal workday (e.g., coaching gymnasts) and 8-h air samples at their residences while doing normal daytime indoor activities (e.g., light house cleaning, viewing television, etc.). Time of collection ranged from 6.07 to 8.41 h, and air volumes ranged from 0.728 to 1.01 m^3^. All participants resided in greater Seattle, WA, USA.

### 2.2. Air Sampling Methods

Air particulates were collected using an AirChek 2000 pump (flow rate 2 L·min^−1^) with an Institute of Occupational Medicine (IOM) Sampler (SKC, Eighty Four, PA, USA) equipped with a stainless steel cassette assembly. The IOM sampler meets air particulate sampling criteria established by the American Conference of Governmental Industrial Hygienists (ACGIH) and the Occupational Safety and Health Administration (OSHA) [25]. The IOM is capable of collecting two size classes of particulates: inhalable particulates and respirable particulates. Inhalable particulates (>4 µm, nominal), which deposit primarily in the upper respiratory tract, were collected with a MultiDust^®^ foam disc (SKC, Eighty Four, PA, USA) of a specific porosity (*D*_50_) of 4 µm (*D*_50_: a particle aerodynamic diameter for which 50% of the particles penetrate) (Figure S1, MultiDust^®^ foam disc Certificate of Conformity, performance criteria 85%, ±10%). This was placed inside the stainless steel cassette assembly, positioned at the IOM inlet. Respirable air particulates (<4 µm, nominal), which are able to penetrate deep inside the lung’s gas-exchange regions, were collected on a 25-mm, 1.0 µm glass fiber filter placed behind the foam disc within the cassette. (Direction of air flow: The air sample was drawn though the IOM sampler by the AirChek 2000 pump, first passing through the MultiDust^®^ foam disc followed by the glass fiber filter. This configuration keeps the sample from contacting the sampling equipment, preventing contamination by the IOM sample housing and pump.). Sampling equipment and cassettes were cleaned and assembled at the laboratory and stored in double sealed plastic bags prior to shipment to the field. After collection, all sample cassettes were placed in SKC’s transportation clip with cover and stored <4 °C in double-sealed plastic bags until analyzed.

The AirChek 2000 pumps were also calibrated at the laboratory prior to shipping. Upon their return, calibrations were verified. All pumps were within manufacturer’s specifications (±5% of set-point). Therefore, no adjustments to recorded field values (e.g., collection times or volumes) were made.

### 2.3. Target Compounds, Extraction, Purification, and Analysis

Air particulate samples (disc and filter) were analyzed for HFRs: Penta-BDEs (BDE-47, -85, -99, -100, and -153); 2-ethylhexyl 2,3,4,5-tetrabromobenzoate (TBB); bis(2-ethylhexyl) 3,4,5,6-tetrabromophthalate (TBPH); and chlorinated organophosphate FRs (ClOPFRs: tris(1,3-dichloro-2-propyl)phosphate (TDCPP or TDCIPP), tris(2-chloroethyl)phosphate (TCEP), and tris(1-chloro-2-propyl)phosphate (TCPP or TCIPP) (Table S1), as described in La Guardia and Hale (2015) [24]. Briefly, disc and glass fiber filters were subjected to accelerated solvent extraction (ASE 200, Dionex, Sunnyvale, CA, USA) with dichloromethane (DCM). Surrogate standards (500 ng of 2,3,4,4′,5,6-hexabromodiphenyl ether (BDE-166); Cambridge Isotope Laboratories, Inc., Andover, MA, USA and 6000 ng of deuterated tris(1,3-dichloro-2-propyl)phosphate (*d*TDCPP); MPI for Biophysical Chemistry, Goettingen, Germany), were added to each sample prior to extraction. Each post-ASE extract was solvent exchanged to hexane, reduced in volume, and added to the top of a solid phase 2-g silica glass extraction column (Isolute, International Sorbent Tech., Hengoed Mid Glamorgan, UK). Columns were eluted with 3.5-mL hexane (fraction 1), followed by 6.5 mL of 60:40 hexane/DCM and 8 mL DCM (fraction 2) and 5 ml 50:50 acetone/DCM (fraction 3). Fraction 2 (containing BFRs) and fraction 3 (containing ClOPFRs) were reduced and transferred to 2 mL maximum recovery vials and reduced to dryness. Samples were reconstituted with 100 μL of methanol containing 800 ng of decachlorodiphenyl ether (DCDE) (AccuStandard, Inc., New Haven, CT, USA) as an internal standard. Analytes in these purified extracts were chromatographically separated by ultra-performance liquid chromatography (UPLC) (Acquity UPLC, Waters Corporation, Milford, MA, USA), operated in the gradient mode (100% methanol (A1) and 100% water (B1)), equipped with a C_18_ UPLC analytical column (Acquity UPLC BEH C_18_, 1.7 µm, 2.1 × 150 mm, Waters Corp.). Analytes were ionized by atmospheric pressure photoionization (APPI), the dopant (acetone) was introduced (150 µL/min) by a liquid chromatography pump (LC-20AD, Shimadzu Corporation, Kyoto, Japan), and product ions were detected by triple quadrupole mass spectrometer (MS) (3200 QTrap, AB Sciex, Framingham, MA, USA) by negative ionization operated in the Multiple Reaction Monitoring (MRM) mode. Quantitation ions for BFRs and BDE-166 were *m/z* 79([^79^Br]^−^) and 81([^81^Br]^−^) and *m/z* 35([^35^Cl]^−^) and 37([^37^Cl]^−^) for ClOPFRs, *d*TDCPP and DCDE, respectively. Quantitation was established using a five-point calibration curve (*R*^2^ > 0.995) for each of the targeted HFRs (analytical standards from AccuStandards, Inc., New Haven, CT, USA). Further details of the UPLC-APPI/MS analysis can be found in Schreder and La Guardia (2014) [26] and La Guardia and Hale (2015) [24].

### 2.4. Quality Control Overview

Complete synopsis of all quality assurance and quality control (QA/QC) procedures and their results are listed within the original manuscripts [23,24]. Briefly, surrogate recoveries for the air samples from the gymnasiums and coaches’ residences from 69% to 97% for BDE-166, from 75% to 121% for *d*TDCPP, and for laboratory blanks, 92% and 75%, respectively. The laboratory blanks did not contain analytes above their limits of detection. Surrogate recoveries for samples collected at the residences/offices were 85% to 115% for BDE-166, 52% to 123% for *d*TDCPP, and 98% and 82% in laboratory blanks respectively. Surrogate recoveries for travel blanks and field samples ranged from 52% to 70% and 61% to 123%, respectively. These blanks also did not contain the targeted analytes above the limits of detection. Each sample was surrogate recovery corrected.

### 2.5. Statistical Analysis

A one-way ANOVA of means with a Tukey’s Honest Significant Difference (HSD*)* post-hoc test was conducted to compare concentrations of ∑ClOPFRs, ∑PBDEs, and ∑TBB + TBPH in the three environments tested. Data were tested for normality and equality of variance, and significance was set at *p* < 0.05. Because normality cannot be reliably checked with small sample size, caution should be used when interpreting these results; however, visual examination of the probability plots did indicate that the data more closely approached a normal distribution without log transformation. Regression analysis was conducted to assess the association between physical chemical properties, specifically log *K_oa_*, and the effect of basing the inhalation exposure estimate on both the inhalable and the respirable particulate fractions. Statistical analyses were performed using Minitab 16.1.0 (Minitab Inc., State College, PA, USA) and Stata v.13 (Stata Corp. LLC, College Station, TX, USA).

## 3. Results and Discussion

### 3.1. Comparison of HFR Air Exposure between Different Indoor Environments

**Figure 1:**
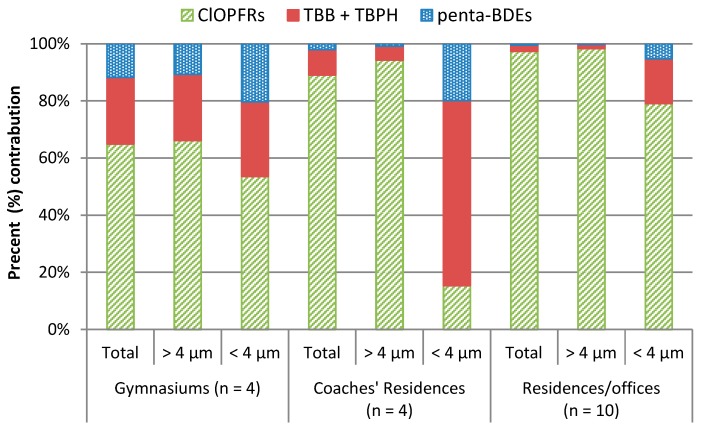
Normalized contributions of various halogenated flame-retardant (HFR) classes to total HFRs (>4 µm + <4 µm), inhalable (>4 µm), and respirable (<4 µm) air particulates within each indoor environment. ClOPFRs, chlorinated organophosphate-FRs; TBB, 2-ethylhexyl 2,3,4,5-tetrabromobenzoate; TBPH, bis(2-ethylhexyl) 3,4,5,6-tetrabromophthalate; penta-BDE, pentabromodiphenyl ether.

**Figure 2:**
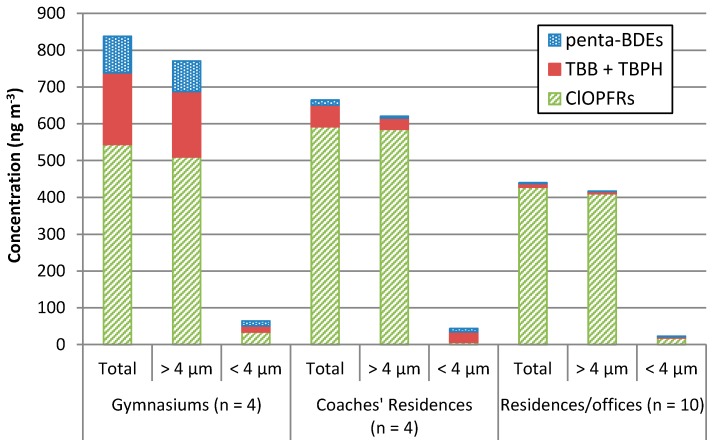
HFR concentrations (ng·m^−3^) of total (>4 µm + <4 µm), inhalable (>4 µm), and respirable (<4 µm) air particulates within each indoor environment.

La Guardia and Hale (2015) [24] observed Penta-BDEs, TBB, TBPH, and ClOPFRs in both respirable (<4 µm, nominal) and inhalable (>4 µm, nominal) air particulate fractions collected at gymnasiums and at the residences of gymnastics coaches using personal exposure air monitors (IOM samplers). Ʃ_inhalable + respirable_ ClOPFRs contributed >65% to the ∑HFR (Ʃ_inhalable + respirable_ HFR: Penta-BDEs, TBB, TBPH, and ClOPFRs) air particulate burdens in the gymnasiums and coaches’ residences (Figure 1). Schreder et al. (2016) [23] also observed ClOPFRs in respirable and inhalable air fractions collected by office-workers wearing IOM samplers during a 24-h work day. Air collection areas included the workers’ homes, offices, and while commuting between their homes and work. ƩClOPFRs were an even greater contributor to the office-workers’ airborne burden, contributing >97% to the ∑HFRs (Figure 1, Penta-BDEs, TBB, and TBPH, results not previously reported, and Table 1). The ∑HFR air burdens within the gymnasiums (835 ng·m^−3^) were 20% greater than at the coaches’ residences (664 ng·m^−3^) and nearly double those collected at the residences/offices (440 ng·m^−3^) (Table 1). The functionality of the indoor space and the amount of HFR-containing products within contribute to indoor HFR dust burdens [1]. This may account for the higher gymnasium HFR air burdens, due to the presence of large amounts of polyurethane foam to reduce impact related injuries [24]. Treated foam products may also form HFR residuals on clothing and personal products used at the gym. These items, if also returned to the coaches’ residences, may have influenced the higher HFR levels observed at their residences compared to levels at the residences/offices. Also, lower HFR air levels observed by Schreder et al. (2016) [23] may be associated with a larger sample size and inclusion of non-residential spaces. However, ClOPFRs were the major contributor to the ∑HFRs in each of the three test environments; their levels were similar (*p >* 0.05) in the three indoor environments (means 544,592 and 428 ng·m^−3^, respectively). The inhalable fraction (>4 µm) carried >92% of the ∑HFRs, while the respirable (<4 µm) carried <8% (Figure 2). Overall, the inhalable ClOPFR fraction contributed >66% to the ∑HFRs. Of the ClOPFRs, TCPP in the inhalable fraction was the major contributor to the ƩClOPFR profile at the coaches’ residences, contributing 91% at the coaches’ residences (536 ng·m^−3^) and 87% (371 ng·m^−3^) in the residence/office samples (Table 1). According to the U.S. EPA Chemical Data Reporting [27]. TCPP production within the U.S. has been on the rise since the mid-1980s. It was equal to that of TDCPP between 1994 and 2006 at 14,000 tonnes·year^−1^, but nearly tripled by 2012 to 38,000 tonnes·year^−1^. TCPP production now exceeds both TCEP and TDCPP, as policies limiting the usage of the latter two compounds have taken effect [23]. However, within the gymnasiums a majority of the polyurethane foam products were installed prior to 2006. Thus, prior market conditions may account for higher TDCPP levels (∑TDCPP, 278 ng·m^−3^) contributing 51%, with TCPP (∑TCPP, 266 ng·m^−3^) contributing nearly half (49%) to the gymnasiums’ ∑ClOPFR profile (Table 1).

Unlike ClOPFRs, constituent profiles of Penta-BDEs, TBB, and TBPH were similar between the gymnasiums, coaches’ residences, and residences/offices. However, their total levels (i.e., respirable + inhalable) at the gymnasiums (mean ∑Penta-BDEs 95.5 ng·m^−3^ and ∑TBB+TBPH 195 ng·m^−3^) substantially exceeded (*p* < 0.05) those detected at the coaches’ residences (13.5 and 58.9 ng·m^−3^, respectively) and in the residence/office environments (1.76, and 9.76 ng·m^−3^, respectively) (Table 1). Total air contributions of the Penta-BDE replacements ∑TBB + TBPH were approximately 2 to 6 times higher than the ∑Penta-BDEs within each of the three environments, with > 52% of the ∑TBB + TBPH air contributions coming from the larger inhalable fraction (Figure 2). The Penta-BDEs and TBB + TBPH inhalable concentrations at the gymnasiums (82.5 and 178 ng·m^−3^, respectively) also exceeded (*p <* 0.05) those detected at both the coaches’ residences (4.84 and 30.6 ng·m^−3^) and residences/offices (0.57 and 6.20 ng·m^−3^) (Figure 2). La Guardia and Hale (2015) [24] observed several Penta-BDEs (i.e., BDE-99, -100, and -153) and TDCPP in the gymnasiums’ respirable (<4 µm) air fraction. These HFRs, plus BDE-47 and -85, were also detected in the gymnasiums’ inhalable air fractions, each at higher concentrations than those detected in the coaches’ residences’ respirable and inhalable air samples. Inhalable levels of BDE-100, TBB, and TDCPP in the gymnasiums were also statistically greater (*p <* 0.05) than levels within the coaches’ residences. A statistical difference was also observed for the respirable Penta-BDEs fraction between the gymnasiums and residences/offices, 13.0 and 1.19 ng·m^−3^, respectively. Given the presence of large amounts of uncovered foam in the gymnasiums, our finding of higher flame retardant concentrations is not surprising, but the size of the data set limits the strength of conclusions that can be drawn. Higher Penta-BDE levels at the gymnasiums may be associated with the historic presence of abundant PBDE-containing products. However, flame-retardants such as those contained in Firemaster 550^®^ (i.e., TBB and TBPH), marketed as a Penta-BDE replacement product, are not only being detected but were found to exceed Penta-BDE levels in these indoor environments. This suggests that replacement HFRs follow the same release and exposure pathways as PBDEs and their toxicological consequences merit evaluation.

### 3.2. HFR Daily Airborne Exposures Equations

The U.S. Centers for Disease Control and Prevention (CDC), Agency for Toxic Substances and Disease Registry (ATSDR) suggests the following equation (Equation (1)) when estimating exposure dose (*D*) (ng·kg^−1^·day^−1^) resulting from contact with contaminated air:

(1) D=(C*IR*AF*EF)/BW

where *C* is the contaminant concentration (ng m^−3^), *IR* is the intake rate of the contaminated medium (16 m^−3^·d^−1^ for adults [28]), *AF* is the bioavailability factor, *EF* is the exposure factor, and *BW* is body weight (70 kg for adults [28]). If exposure is intermittent or irregular, the *EF* can then be calculated to average the doses over the exposure interval. In many instances, *EF* will equal 1, representing a constant exposure to the contaminant. This approach was adapted by Nouwen et al. (2001) [29] to calculate daily health risk to dioxin air emissions from municipal waste incinerators, assuming an *AF* of 100%. They also replaced *EF* with an alveolar fraction (*fr*) default value of 0.75; i.e., 75% of the inhaled contaminant will be retained and taken up in the lung’s alveolar region (Equation (2)).

(2) $$D_{fr}=\left(C*IR*AF*fr\right)/BW$$

Using this approach, Chen et al. (2011) [30] assessed PBDE in the atmosphere at an e-waste recycling site and observed inhalation exposure exceeded the maximum allowed daily intake level of 0.26 ng·kg^−1^·d^−1^ for BDE-99, established by the Dutch National Institute for Public Health and the Environment (RIVM) [31]. However, contributions from inhaled particulates may influence these estimates, as the size of the airborne particulate matter (PM) will affect its fate within the respiratory tract, thereby affecting bioavailability of associated contaminants. PM with aerodynamic diameters <2.5 µm (PM_2.5_) can penetrate deep inside the lung’s gas exchange region, while larger sized PM may reside in the head or tracheobronchial region of the respiratory tract [32]. Lyu et al. (2016) [33] collected size-resolved air particulates (<0.4 to >9.0 µm) at an urban site in Shanghai, China, between 2012 and 2013. Deposition fluxes indicated that only 11% of the PBDE-laden particles would likely enter the alveolar region. Yang et al. (2014) [34] suggested that >50% of organophosphate flame-retardants were associated with suspended particulate matter of a size indicating deposition in the lung’s head region, “indicating a comparatively lower exposure risk” (p. 69). Our previous reports indicated that >92% of the flame-retardant airborne burden was associated with larger particulates (>4 µm) [23,24]. It follows that not only should exposure via the lungs’ alveolar exchange region be considered when evaluating health risk exposure to air particulates, but also exposure resulting from swallowing the non-respirable fraction and its subsequent uptake through the digestive tract. This more comprehensive approach is also supported by the European Commissions’ (EC) Registration, Evaluation, Authorization and Restriction of Chemical (REACH) program [35].

The bioavailability of several PBDEs in the digestive tract was previously evaluated by feeding Sprague-Dawley rats PBDE-laden house dust Standard Reference Material (SRM) 2585 (National Institute of Standards and Technology (NIST), Gaithersburg, MD, USA). Results indicated a >80% absorption efficiency for PBDE with six or less bromines, down to approximately 40% efficiency for the full brominated BDE-209 [36]. Fang et al. (2014) [37] also evaluated the bioavailability of 20 flame retardants in SRM 2585 and 17 house dust samples collected between 2006 and 2010 using an in vitro Tenax bead-assisted sorptive method. Tenax beads have been validated as an effective material to evaluate bioaccessibility of polyaromatic hydrocarbons (PAHs) and pesticides in soils and sediments [38]. Using Tenax beads and simulated digestive fluids, Fang et al. (2014) [37] observed a >80% bioaccessibility for the ClOPFRs, >55% for PBDEs with six or less bromines (35% for BDE-209), and <50% for TBB (49%) and TBPH (29%) (Table S1). We created an exposure estimate using these values: the inhalable air fraction (*AF)* was substituted with the mean digestive tract bioaccessibility (*fd*) factor (Table S1), as suggested by Fang et al. (2014) [37] (*D_inh_*, Equation (3)). This assumed these larger air particulates will be trapped on the mucosa lining of the respiratory tract, swallowed, and absorbed through the digestive tract. To represent the bioavailability factor (*AF*) of the respirable air particulates entering the alveolar gas-exchange region of the lung, a value of 100% was assumed for equation *D_res_ (*Equation (4)). Both exposure routes (*D_res_*, *D_inh_*) are then combined to estimate the total air exposure (*D_r+i_*) resulting from uptake through the alveoli and digestive tract (Equation (5)).

Inhalable (>4 µm) air exposure (*D_inh_*):

(3) $$D_{\mathrm{inh}}=\left(C*IR*fd*EF\right)/BW$$

Respirable (<4 µm) air exposure (*D_res_*):

(4) $$D_{\mathrm{res}}=\left(C*IR*AF*EF\right)/BW$$

Total (<4 µm + >4 µm) air exposure *(D_r+i_)*:

(5) $$D_{r+i}\;=\;D_{\mathrm{res}}\;+\;D_{\mathrm{inh}}$$

### 3.3. Analysis of HFR Daily Airborne Exposures, Derived by Alveolar and Digestive Tract Bioavailability

**Figure 3:**
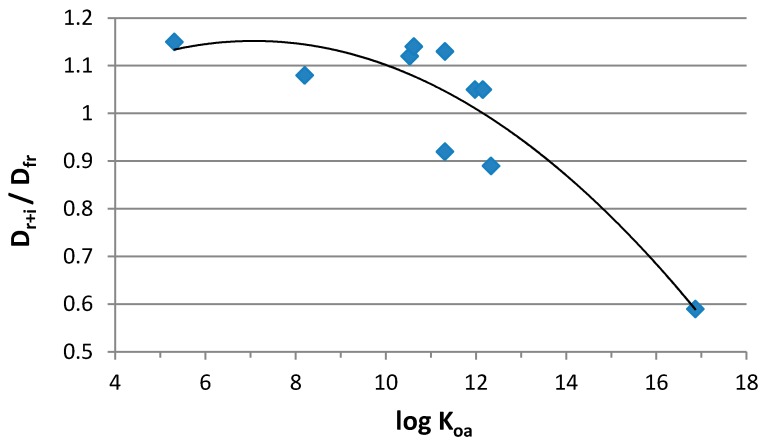
Association of daily inhalation dose exposure comparisons (*D_r+i_/D_fr_*) with log octanol-air partitioning coefficient (*K_oa_*). *D_r+i_* = ∑respirable + inhalable fraction (Equation (5)). *D_fr_* = alveoli fraction (Equation (2)). Regression analysis, curvilinear relationship (*p* = 0.007).

Using the Nouwen et al. (2001) [29] equation (Equation (2)), daily airborne exposures were first evaluated for each HFR in the three indoor environments (i.e., gymnasiums, coaches’ residences, and residences/offices) previously sampled by La Guardia and Hale (2015) [24] and Schreder et al. (2016) [23]. These values were then compared to exposure values prepared by the total respirable + inhalable air exposure equation (*D_r+i_*) (Equation (5)) (Table 2). All exposures were evaluated based on a 24-h exposure rate (*EF*, default value of 1) using the mean air concentration (*C*) for each of the detected HFRs. Using the total air exposure equation (Equation (5)), the average multiple uptake exposure rates (*D_r+i_*) of the three indoor environments for both Penta-BDEs and ClOPFRs were estimated to be an average of 10% higher (ratios of *D_r+i_/D_fr_* ranged between 0.96–1.17 and 1.09–1.11, respectively (Table 2)) compared to values derived from the alveolar fraction (*D_fr_*) alone. In contrast, exposure to the more hydrophobic HFRs, TBB + TBPH was 20% lower with the *D_r+i_* approach, (*D_r+i_/D_fr_* range 0.66–0.94, Table 2). The most significant difference was for TBPH, which was 41% less on average compared to values derived from the alveolar fraction used by Nouwen et al. (2001) [29] and Chen et al. (2011) [30] (Equation (2)). Because physical chemical properties, in particular log octanol-air partitioning coefficient (*K_oa_*), can be expected to relate to particle partitioning, we assessed the relationship between the *D_r+i_/D_fr_* ratio and log *K_oa_*. With the revised *D_r+i_* formula, a general increase in estimated exposure levels was observed for HFRs with a log *K_oa_* < 12, while a decrease was observed for those with a log *K_oa_* > 12 (Figure 3). Regression analysis showed a strong curvilinear association (*p =* 0.007), with a lower ratio among compounds with higher log *K_oa_*.

Daily exposures, as estimated by the revised *D_r+i_* formula, were still dominated by the ClOPFRs, *D_r+i_* range 79.9–111 ng·kg^−1^·d^−1^. Penta-BDE exposure at the gymnasiums (15.7 ng·kg^−1^·d^−1^) was 5 times higher than at the coaches’ residences (2.72 ng·kg^−1^·d^−1^) and >40 times higher than the residences/offices (0.36 ng·kg^−1^·d^−1^) (Table 2). Exposure to the Penta-BDE replacements TBB + TBPH via air particulates exceeded those of the Penta-BDEs at both the residences/offices (1.35 and 9.45 ng·kg^−1^·d^−1^, respectively) and the coaches’ residences (0.36 and 2.72 ng·kg^−1^·d^−1^, respectively (Table 2)). Expected total intakes of individual HFRs expressed by either exposure equation were below their corresponding chronic oral reference dose (R*f*D) for PBDEs established by the U.S. EPA [39], TBB, TBPH, and ClOPFRs published by Hardy et al. (2008) [40] and Ali et al. (2012) [41], respectively (Table S2). However, the U.S. EPA recommended R*f*D values are based on relatively old toxicological studies, and other studies have suggested lower R*f*D standards for BDE-99 (0.26 ng·kg^−1^·d^−1^) [31]. Half of this value was detected at the residences/offices (*D_r+i_*, 0.15 ng·kg^−1^·d^−1^) and was exceeded 3 to 25 times within the coaches’ residences and gymnasiums, with respective estimates of 0.89 and 6.53 ng·kg^−1^·d^−1^. The use of HFRs in consumer products may be declining after the passage of California’s revised TB 117-2013 [4], which modified flammability standards for upholstered furniture. However, other products in the indoor environment contribute to flame retardant exposure. For example, building products can also contain HFRs at percent by weight (e.g., TCPP in spray foam insulation and hexabromocyclododecane (HBCD) in insulation boards), these are not covered by this new regulation. These treated HFR-materials will remain in usage for decades, and may increase in abundance as more efficient building insulation is needed to further reduce the use of greenhouse gas emitting fossil fuels for heating. In addition, electronics products typically contain percent levels of flame retardants and also contribute to indoor exposures [42,43].

## 4. Conclusions

In these studies, a wide range of former and currently marketed HFRs used to treat polyurethane foam was detected in indoor air. Two particulate air fractions (respirable: <4 µm and inhalable: >4 µm) were collected during each sampling. Multiple HFRs were detected in both fractions. The inhalable fraction carried the bulk of the ∑HFRs, with ∑ClOPFR contributing 65% to 97% to the total. ∑TBB + TBPH air contributions were approximately 2 to 6 times higher than the ∑Penta-BDEs within each indoor environment, indicating that these Penta-alternatives are also escaping products and entering the breathing zone in indoor environments. The gymnasiums’ ∑Penta-BDEs and ∑TBB + TBPH inhalable concentrations exceeded those detected at the coaches’ residences and residences/offices. Individual flame retardant levels of BDE-100, TBB, and TDCPP were also significantly higher. A statistical difference was also observed for the respirable Penta-BDEs fraction between the gymnasiums and residences/offices. In our exposure analysis considering airborne particulate size, deposition site, and HFR bioavailability, we found that a refined model creating separate estimates for exposure to smaller particulates, via the lung, and larger particulates, via ingestion, altered total inhalation exposure estimates. Estimated daily inhalation exposures were greater for HFRs with a log *K_oa_* < 12 (i.e., Penta-BDEs and ClOPFRs) and lower for those with a log *K_oa_* > 12 (i.e., TBB and TBPH). The most significant change was for TBPH, averaging a 41% decrease in bioavailability. Although inhalation exposure levels did not exceed lowest observed adverse effects levels established by the U.S. EPA, inhalation is just one contributor to total exposure, and some have suggested more stringent standards. These lower exposure levels were exceeded at the coaches’ residences and the gymnasiums for BDE-99. Therefore, future work should continue to focus on uptake exposure and health effects of HFRs, as new and recycled HFRs continue to be introduced into consumer products with the potential to be released to the indoor environment throughout the products’ life-cycles.

## Figures and Tables

**Table 1:** Halogenated flame retardants (HFRs) inhalable (>4 µm) and respirable (<4 µm) air particulate fraction concentrations (ng·m^−3^) of each indoor environment.

	^1^ Gymnasiums (n = 4)				^1^ Coaches’ Residences (n = 4)				^2^ Residences/Offices (n = 10)			
	Inhalable (>4 µm)		Respirable (<4 µm)		Inhalable (>4 µm)		Respirable (<4 µm)		Inhalable (>4 µm)		Respirable (<4 µm)	
HFRs	Range (%RD)	Mean ^3^	Range (%RD)	Mean ^3^	Range (%RD)	Mean ^3^	Range (%RD)	Mean ^3^	Range (%RD)	Mean ^3^	Range (%RD)	Mean ^3^
BDE-47	10.1–82.7 (100%)	33.0	nd–5.70 (75%)	3.06	0.20–2.30 (100%)	1.45	nd–8.50 (75%)	3.34	nd–1.17 (40%)	0.23	nd–1.39 (40%)	0.22
BDE-85	1.50–3.00 (100%)	2.18	nd (0%)	0.05	nd–2.00 (25%)	0.54	nd (0%)	0.05	nd (0%)	0.05	nd (0%)	0.05
BDE-100	4.40–15.4 (100%)	8.03	0.60–5.50 (100%)	2.38	0.50–1.90 (100%)	1.35	nd–1.60 (75%)	1.04	nd–0.19 (20%)	0.07	nd–1.02 (40%)	0.32
BDE-99	18.5–58.5 (100%)	35.4	nd–9.60 (75%)	5.54	nd–4.30 (50%)	1.23	nd–11.1 (50%)	3.10	nd–0.39 (50%)	0.17	nd–1.59 (70%)	0.55
BDE-153	1.30–6.10 (100%)	3.90	nd–6.70 (50%)	1.95	nd–0.70 (50%)	0.28	nd–3.00 (50%)	1.15	nd (0%)	0.05	nd (0%)	0.05
Penta-BDE	36.9–164 (100%)	82.5	1.80–20.7 (100%)	13.0	3.50–7.10 (100%)	4.84	nd–24.1 (75%)	8.68	nd–1.54 (70%)	0.57	nd–3.74 (70%)	1.19
∑Penta-BDE ^4^	95.5				13.5				1.76			
TBB	46.4–220 (100%)	143	nd–20.2 (75%)	11.4	2.60–55.4 (100%)	22.0	nd–55.2 (75%)	21.4	nd–23.3 (30%)	3.23	nd–18.4 (40%)	2.99
TBPH	4.90–71.9 (100%)	34.3	nd–12.8 (75%)	5.41	nd–18.3 (75%)	8.61	nd–18.6 (50%)	6.93	nd–25.6 (20%)	2.97	nd–3.44 (50%)	0.57
TBB + TBPH	51.3–184 (100%)	178	nd–31.0 (75%)	16.8	2.60–73.7 (100%)	30.6	nd–73.8 (75%)	28.3	nd–48.9 (30%)	6.20	nd–21.9 (50%)	3.56
∑TBB + TBPH ^4^	195				58.9				9.76			
TCEP	nd (0%)	0.05	nd (0%)	0.05	nd (0%)	0.05	nd (0%)	0.05	nd–77.8 (89%)	19.1	nd (0%)	0.75
TCPP	136–525 (100%)	266	nd (0%)	0.05	209–1360 (100%)	536	nd (0%)	0.05	16.0-1180 (100%)	371	nd–28.6 (60%)	12.3
TDCPP	125–397 (100%)	244	32.0–36.5 (100%)	34.3	32.0–69.2 (100%)	50.1	nd–26.4 (25%)	6.64	nd–82.2 (33%)	19.1	nd–20.9 (50%)	4.97
ClOPFRs	376–650 (100%)	510	32.0–36.5 (100%)	34.4	241–1420 (100%)	585	nd–26.4 (25%)	6.74	61.1–1180 (100%)	410	nd–34.4 (80%)	18.0
∑ClOPFRs ^4^	544				592				428			
HFRs	608–888 (100%)	771	33.8–83.0 (100%)	64.2	272–1500 (100%)	620	nd–124 (75%)	43.8	61.5–1180 (100%)	417	5.80–37.8 (100%)	22.7
∑HFRs ^4^	835				664				440			

^1^ La Guardia, M.J.; R. C. Hale 2015 [24]. Halogenated flame-retardant concentrations in settled dust, respirable and inhalable particulates and polyurethane foam at gymnastic training facilities and residences. *Environ. Int. 79*, 106–114. ^2^ Schreder, E. D.; N. Uding; M. J. La Guardia 2016 [23]. Inhalation a significant route for chlorinated organophosphate flame retardants. *Chemosphere 64*, 181–186. ^3^ Values reported as non-detect (nd) were replaced with ½ of the reported detection limit when calculating mean values for statistic evaluation. ^4^ Sum of the inhalable + respirable air particulate fractions. %RD = % rate of detection.

**Table 2:** Daily inhalation exposure dose by alveoli fraction (*D_fr_*) and alveoli plus digestive tract (*D_r+i_*) (ng·kg^−1^·bw·d^−1^) and their ratio (*D_r+i_/D_fr_*).

	Gymnasiums (n = 4)			Coaches’ Residences (n = 4)			Residences/Offices (n = 10)			*D_r+i_/D_fr_*
Analytes	*D_fr_*	*D_r+i_*	*D_r+i_/D_fr_*	*D_fr_*	*D_r+i_*	*D_r+i_/D_fr_*	*D_fr_*	*D_r+i_*	*D_r+i_/D_fr_*	Mean (n = 3)
BDE-47	6.18	6.21	1.00	0.82	1.01	1.23	0.08	0.09	1.13	1.12
BDE-85	0.38	0.33	0.87	0.10	0.09	0.90	0.02	0.02	1.00	0.92
BDE-100	1.78	1.70	0.96	0.41	0.43	1.05	0.07	0.08	1.14	1.05
BDE-99	7.02	6.53	0.93	0.74	0.89	1.20	0.12	0.15	1.25	1.13
BDE-153	1.00	0.94	0.94	0.25	0.30	1.20	0.02	0.02	1.00	1.05
Penta-BDEs	16.4	15.7	0.96	2.32	2.72	1.17	0.31	0.36	1.16	1.10
TBB	26.5	18.6	0.70	7.44	7.36	0.99	1.07	1.05	0.98	0.89
TBPH	6.81	3.27	0.48	2.66	2.10	0.79	0.61	0.31	0.51	0.59
TBB + TBPH	33.3	21.9	0.66	10.1	9.45	0.94	1.67	1.35	0.81	0.80
TCEP	0.26	0.31	1.19	0.26	0.31	1.19	3.40	3.66	1.08	1.15
TCPP	45.7	49.4	1.08	92.0	99.4	1.08	65.7	71.5	1.09	1.08
TDCPP	47.7	54.1	1.13	9.82	11.1	1.13	4.13	4.76	1.15	1.14
ClOPFRs	93.7	104	1.11	102	111	1.09	73.3	79.9	1.09	1.10
^1^∑HFRs	143	141	0.99	115	123	1.07	75.2	81.6	1.09	1.05

^1^ ∑HFRs = Penta-BDEs + TBB + TBPH + ClOPFRs.

## Supplementary Materials

The following are available online at www.mdpi.com/1660-4601/14/5/507/s1, Table S1: HFR nomenclature, octanol air partitioning coefficients (*K_oa_*) *, digestive tract bioaccessibility ^†^, and air particle sorption models ^‡^, Table S2: HFR corresponding chronic oral reference dose (*RfD*) values, ng·kg^−1^·d^−1^. Figure S1: MultiDust^®^ foam disc Certificate of Conformity, performance criteria 85%, ±10%.
