# Supplementary material for: Human Indoor Exposure to Airborne Halogenated Flame Retardants: Influence of Airborne Particle Size
Source: Int J Environ Res Public Health. 2017 May 9;14(5):507. doi: 10.3390/ijerph14050507 (PMC5451958; doi:10.3390/ijerph14050507)
Supplement: Supplementary file 1 [file ijerph-14-00507-s001.pdf]

Human Indoor Exposure to Airborne Halogenated Flame Retardants: Influence of Airborne Particle Size

Mark J. La Guardia*, Erika D. Schreder, Nancy Uding and Robert C. Hale

Table S1, HFR nomenclature, octanol air partitioning coefficients (K_{oa})*, digestive tract bioaccessibility† and air particle sorption models‡.

Analyte	CAS#	Compound	log K_{oa}*	Digestive tract bioaccessibility fraction, *(fd)* (mean, n=17 †)	Fraction sorbed to airborne particles (phi), models‡		
					K_{oa}‡	MacKay ‡	Junge-Pankow ‡
BDE-47	5436-43-1	2, 2′, 4, 4′-tetrabromodiphenyl ether	10.53	0.73	0.533	0.34	0.4
BDE-85	182346-21-0	2, 2′, 3, 4, 4′-pentabromodiphenyl ether	11.66	0.63	0.873	0.756	0.9
BDE-99	60348-60-9	2, 2′, 4, 4′, 5-pentabromodiphenyl ether	11.31	0.65	0.983	0.963	0.8
BDE-100	189084-64-8	2, 2′, 4, 4′, 6-pentabromodiphenyl ether	11.977	0.63	0.625	0.429	0.949
BDE-153	68631-49-2	2, 2′, 4, 4′, 5, 5′-hexabromodiphenyl ether	12.15	0.55	0.906	0.814	0.965
TBB (EH-TBB)	183658-27-7	2-ethylhexyl 2, 3, 4, 5-tetrabromobenzoate	12.335	0.49	0.581	0.385	0.977
TBPH (BEH-TEBP)	26040-51-7	2-ethylhexyl 2, 3, 4, 5-tetrabromophthalate	16.864	0.26	0.999	0.997	1
TCEP	115-96-8	tris (2-chloroethyl) phosphate	5.311	0.80	2.94E-05	1.33E-05	4.02E-06
TCPP (TCIPP)	13674-84-5	tris (1-chloro-2-propyl) phosphate	8.203	0.81	0.0309	0.0142	0.00312
TDCPP (TDCIPP)	13674-87-8	tris (1,3-dichloro-2-propyl) phosphate	10.622	0.83	0.858	0.732	0.451

*, ‡ USEPA's EPI Suite (EPIWEB 4.1), Sorption to Aerosols (25 deg C) (AEROWIN V1.00)

†Mean (n=17) digestive tract bioaccessibility values from: Fang M. and Stapleton, H. M. 2014 Evaluating the bioaccessibility of flame retardants in house dust using an In vitro tenax bead-assisted sorptive physiologically based method. Environ. Sci. Technol. 48, 13323 -13330.

Table S2, HFR corresponding chronic oral reference dose (*RfD*) values, ng kg^{-1} d^{-1}

Analyte	*RfD*	Reference
2, 2′, 4, 4′-tetrabromodiphenyl ether (BDE-47)	† 100	† USEPA, 2014b, Technical Fact Sheet – PBDEs and PBBs<br>‡ Winter-Sorkina et al., 2006 RIVM-report 320100002
2, 2′, 4, 4′, 5-pentabromodiphenyl ether (BDE-99)	† 100, (‡0.26)	
2, 2′, 4, 4′, 5, 5′-hexabromodiphenyl ether (BDE-153)	† 200	
2-ethylhexyl 2, 3, 4, 5-tetrabromobenzoate (TBB or EH-TBB)	*20,000	*Hardy et al., Environ. Sci. Technol. 42 (2008) 9453 -9454

2-ethylhexyl 2, 3, 4, 5-tetrabromophthalate (TBPH or BEH-TEBP)	[*]20,000	
tris (2-chloroethyl) phosphate (TCEP)	[₮]22,000	
tris (1-chloro-2-propyl) phosphate (TCPP or TCiPP)	[₮]80,000	[₮]N. Ali et al., Chemosphere 88 (2012) 1276 -1282
tris (1,3-dichloro-2-propyl) phosphate (TDCPP or TDCIPP)	[₮]15,000	

[†] USEPA, 2014b, Technical Fact Sheet – Polybrominated Diphenyl Ethers (PBDEs) and Polybrominated Biphenyls (PBBs) United States Environmental Protection Agency, Washington, D.C., United States https://www.epa.gov/sites/production/files/2014-03/documents/ffrrofactsheet_contaminant_perchlorate_january2014_final_0.pdf (accessed March, 2017)

[‡] Winter-Sorkina, R. D., Bakker, M. I., Wolterink, G., Zeilmaker, M. J., 2006. Brominated flame retardants; occurrence, dietary intake and risk assessment, Dutch National Institute for Public Health and the Environment (RIVM), RIVM-report 320100002.

Certificate of Conformity

Testing carried out according to BSEN 13205 (2002)
Foam insert for the respirable fraction EN481 (1993)
Tested at the Health & Safety Laboratory,
Harpur Hill, Buxton SK17 9JN

2nd July 2010

HEALTH & SAFETY LABORATORY

www.hsl.gov.uk

D50 Cut Off Particle Size

ID.	D50 (µm)	Dia (mm)	Len (mm)
REC1	4.76	15.84	12.1
REC1	4.87	15.92	11.9
REC1	4.94	15.91	11.75
REC1	4.99	16.01	11.7
REC1	5.07	15.92	11.71
REC1	4.89	15.68	11.96
REC1	5.1	15.79	11.75
REC1	5	15.85	11.82
REC1	4.91	15.87	11.79
REC1	4.89	15.79	11.08
Mean	4.94	15.85	11.76
SD	0.10	0.09	0.26
Target	4.25		

Sample flow rate: 2 l/min

Bias Map
Foam Insert Batch Reference:
REC

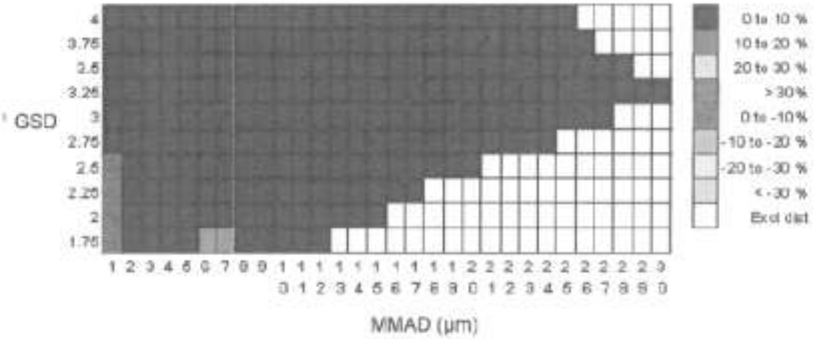

Measured Mean Penetration

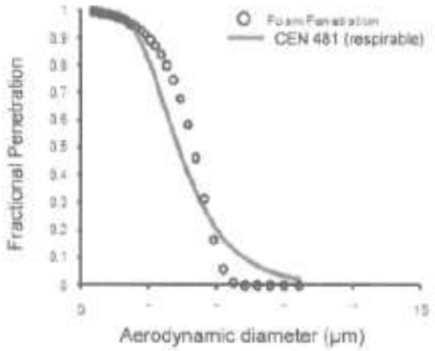

Bias Performance Criteria (BPC): 85% or more
of all the calculated biases are within +/- 10%
CONFORMS

Signed: a. Thorpe

Date: 7/7/10

This certificate of conformity indicates that the above foam
inserts were tested at the above establishment on the designated date and that the foam inserts complied with the Bias Performance Criteria of BSEN 13205 in which 85% of the calculated biases are within +/- 10%

Figure S1, MultiDust® foam disc Certificate of Conformity, performance criteria 85%, ±10 %.
